# Psychiatric morbidity and gambling disorder: A systematic review and meta-analysis of population-based surveys

**DOI:** 10.1192/j.eurpsy.2025.10122

**Published:** 2025-10-23

**Authors:** Gian M. Galeazzi, Mattia Marchi, Augusto C. Castagnini

**Affiliations:** 1 Department of Biomedical, Metabolical and Neural Sciences, University of Modena and Reggio Emilia, Modena, Italy; 2 Department of Mental Health and Pathological Addictions, Azienda USL-IRCCS di Reggio Emilia, Reggio Emilia, Italy

**Keywords:** comorbidity, DSM, epidemiology, gambling, ICD, prevalence

## Abstract

**Background:**

The aim of this study is to determine the prevalence and type of mental disorders associated with pathological gambling/gambling disorder (GD) in the general population.

**Methods:**

Systematic review and meta-analysis of adult population-based studies reporting on psychiatric comorbidity of GD according to International Classification of Diseases (ICD-10/ICD-11), Diagnostic and Statistical Manual of Mental Disorders (DSM-IV/DSM-5) criteria, or widely used assessment instruments. PubMed, Scopus, and Web of Science databases were searched for relevant studies in English. The study’s protocol was preregistered in PROSPERO (CRD42024574210).

**Results:**

Of 454 articles published between 1993 and 2024, 12 met the inclusion criteria. Most studies used DSM-IV or DSM-5 criteria (only two ICD-10 criteria), and were evenly distributed across Europe, North America, and Southeast Asia. The weighted average prevalence of any mental disorder in individuals with GD was 82.2%. High comorbidity rates were found for substance use disorders (SUDs) (34.2%), mood disorders (30.9%), and anxiety disorders (29.9%), followed by personality (14.3%) and psychotic (5.9%) disorders. Meta-analysis indicates that individuals with GD are 10.7 (95% confidence interval [CI]: 5.7;20.1) times more likely to develop any mental disorder than the general population. The odds ratio for mental disorders associated with GD were 5–12 times higher for nicotine dependence, drug use disorder, alcohol use disorder, and SUD, and 3–4 times higher for anxiety and mood disorders.

**Conclusions:**

These findings add weight to the view that GD is associated with a significantly increased risk for addictive behaviors, mood, and anxiety disorders.

## Introduction

Gambling disorder (GD) as defined by current psychiatric classifications, such as the American Psychiatric Association’s Diagnostic and Statistical Manual of Mental Disorders, fifth edition (DSM-5) [1], and the World Health Organization ICD-11 Classification of Mental, Behavioral, and Neurodevelopmental Disorders (ICD-11) [2], involves a persistent pattern of gambling behavior leading to significant clinical distress and impairment in personal, family, social, or other areas of functioning over a period of at least 12 months.

Drawing on similarities in symptom pattern and neurobiological features, the DSM-5 [1] has classified GD under “substance-related and addictive disorders,” while DSM-IV [3] listed “pathological gambling” within “impulse control disorders.” The DSM-5 moved a step toward a dimensional classification of gambling behavior, being defined in degree of severity as mild, moderate, or severe based on the number of symptoms. The criteria required for diagnosing GD have become less restrictive: the threshold has been reduced from five to four criteria, and the “illicit acts” item has been withdrawn from the symptom checklist, since it also plays a role in the diagnosis of antisocial personality disorder. Exclusion criteria, such as manic episode, have remained almost unchanged.

The ICD-10 [4] was developed in parallel with DSM-IV and included “pathological gambling” under “habit and impulse disorders.” In ICD-11 [2], it has been renamed “gambling disorder” and subsumed along with “gaming disorder” within the newly proposed category of “disorders due to addictive behaviors.” Differential diagnosis of GD involves bipolar disorder, obsessive-compulsive disorder, substance use disorders (SUDs), and the effects of medications, in addition to hazardous gambling or betting.

In keeping with findings of major psychiatric epidemiological surveys, GD remains a relatively rare condition with a lifetime prevalence higher in men than in women [5, 6]. It is also reported to be associated with increased risk for DSM-IV substance use, anxiety, mood, and personality or impulse control disorders [5–8]. Comorbidity patterns are likely to differ by gender, since substance abuse shows a characteristic male prevalence, while depression and anxiety disorders are more common in women [9]. In addition, there seems to be a particular compliance problem in cases with GD and comorbid mental disorders, which may have adverse effects on treatment and outcome [10].

Previous reviews [11] were mainly based on population-based studies using DSM-III-R or DSM-IV criteria, while little is known about psychiatric morbidity associated with GD according to current classifications of mental disorders, especially ICD revisions. An updated review of the literature is, therefore, all the more necessary to enhance the evidence base on this topic, yet detailed consideration of the prevalence of GD falls beyond the scope of the present review.

## Methods

The guidelines provided by the Preferred Reporting Items for Systematic Reviews and Meta-Analyses (PRISMA) 2020 statement were followed [12]. The study’s protocol was preregistered in the International Prospective Register of Systematic Reviews (PROSPERO n. CRD42024574210).

### Search strategy and selection criteria

To identify relevant studies reporting on psychiatric comorbidity of GD, PubMed, Scopus, and Web of Science databases were searched using the following terms: ((gambling) and (“ICD” or ICD-10 or ICD-11 or “DSM” or DSM-IV or DSM-5) and (epidemiology or comorbidity or prevalence) and adult)) (see Supplementary Table 1 for the search strings used).

The titles and abstracts of all articles meeting the search criteria were reviewed, and full-text articles were retrieved and assessed for inclusion in the study. Additional articles were identified from the reference list of eligible publications and review articles. Studies were selected if they: (a) assessed the prevalence and type of mental disorders associated with GD according to ICD and DSM criteria or widely used diagnostic instruments; (b) were representative adult population samplings (i.e., aged 18 years and over), whether random selected or treating-seeking in- and outpatient mental health services; and (c) were written in English and published from January 1, 1993 to July 31, 2025. Psychiatric comorbidity was defined as follows: (1) SUDs (i.e., alcohol, drug, tobacco, etc.); (2) psychotic disorders (schizophrenia, schizoaffective disorder, delusional disorder, and brief psychotic disorder); (3) mood (affective) disorders (manic disorder, bipolar disorder, depressive disorder, and recurrent depressive disorder); (4) neurotic/anxiety disorders (panic, phobic, and generalized anxiety disorders [GADs]) obsessive-compulsive disorder, stress-related and adjustment disorders; (5) personality disorders and habit and/or impulse control disorders; (6) any ICD or DSM category, excluding “pathological gambling” (i.e., DSM-IV/DSM-IV-TR 312.31 or ICD-10 F63 code) [3, 4], or “GD” (DSM-5 312.31 and ICD-11 6C50 code) [1, 2]. Where publications reported overlapping data, the most informative or recent were selected.

Exclusion criteria were as follows: (a) other languages than English; (b) adolescents under 18 years; (c) clinical trials, family, and case–control studies of individuals with gambling behavior and comorbid mental disorders; (d) studies of defined subgroups of the population (i.e., young adults, men or women, in- or out-patients, ethnic groups, etc.).

### Data collection

Two authors independently extracted relevant data from selected studies including: (a) general characteristics (i.e., author, year of publication, country, and sampling method); and (b) demographic and clinical features (sample size, age, sex distribution, socioeconomic status, number and percentage [%] of cases with GD, number [%] of controls, and number [%] and type of ICD or DSM diagnoses among gamblers and controls). Extraction sheets of each study were compared for consistency, and disagreements were discussed and resolved via consensus. When not reported, data were derived from information available in the articles. Otherwise, corresponding authors were contacted to obtain missing information.

### Study quality

The risk of bias was assessed by means of the Joanna Briggs Institute (JBI) critical appraisal checklist for prevalence studies [13]. This checklist consists of nine items encompassing sample characteristics, case identification and ascertainment, statistical analysis, and response rate. For each study included in the review, the overall risk of bias was rated on a 9-point scale as high (0–3), moderate (4–6), or low (7–9).

### Statistical analysis

The weighted average prevalence of mental disorders associated with GD was calculated by pooling the raw prevalence of individual studies using the number of cases with GD as a weighted factor. In addition, Mantel–Haenszel combined odds ratio (OR) with 95% confidence interval (95% CI) was used to estimate the association of GD and comorbid mental disorders compared with the general population. Heterogeneity across studies was reported using *I* ^2^ statistics [14]. *I* ^2^ value ranges from 0–25%, 26–50%, 51–75% to 76–100% and means low, moderate, substantial, and high heterogeneity, respectively. Where indicated, meta-analysis and meta-regression were conducted for the above-defined diagnostic categories using the following moderator variables: year (before/after 2013 DSM-5 publication), geographical area, sampling method (random selected vs. treating-seeking sample), mean age, female %, ICD, or DSM diagnosis.

All statistical analyses were performed with R software, version 4.4.0 [15], using meta and metaphor packages [16, 17]. Statistical significance was evaluated at the 0.05 level with two-sided tests.

### Changes to PROSPERO protocol

The JBI critical appraisal checklist for prevalence studies [13] was introduced to assess the risk of bias of individual studies, whereas publication bias was not examined because the meta-analyses being conducted included fewer than 10 studies. The small number of population-based studies using ICD-10 criteria published to date also makes meaningful comparison with DSM based findings difficult. Furthermore, leave-one-out analyses were undertaken to determine the robustness of results against potential outlier effects.

## Results

### Study characteristics

**Figure 1.**
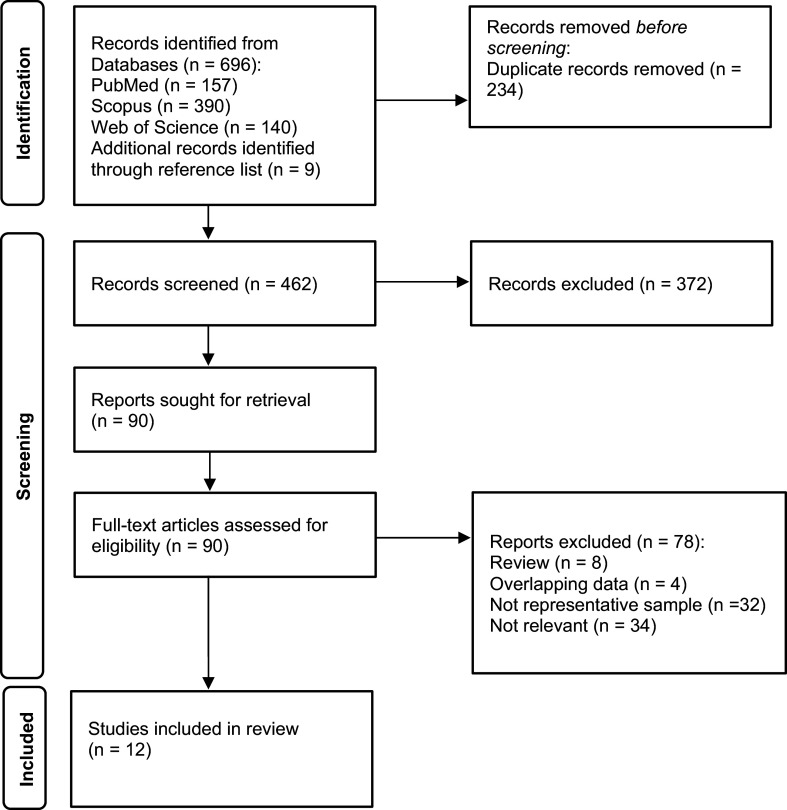
PRISMA flow chart of reviewed articles.

After removal of duplicates, 462 articles published between January 1993 and July 2025 were identified, and 12 met the inclusion criteria (Figure 1, PRISMA flow chart).

There were 10 large-scale population-based surveys reporting on psychiatric comorbidity of GD using randomly selected samples [5-8, 18–23] and 2 nationwide treatment-seeking samples from community mental health services [24, 25]. Nearly all included studies were conducted in high-income countries and were evenly distributed across Europe, North America, and Southeast Asia. The most widely employed checklist for diagnosing GD was the DSM-IV [3] and DSM-5 [1] criteria, followed by the ICD-10 criteria [4], the Problem Gambling Severity Index [26], and the South Oaks Gambling Screen [27]. The reported lifetime prevalence of GD ranged from 0.4 to 2.7% according to DSM criteria [5-8, 18, 20, 21] with higher rates observed among men and in Southeast Asia than in Europe or North America (Table 1).

**Table 1.** Outlook of population-based studies reporting on psychiatric comorbidity of gambling

Author	Study	Diagnosis	Main findings	Risk of bias^a^
Welte et al. [18]	USA CATI 1999–2000	SOGS; DIS/DSM-IV	PG lifetime prevalence 2.0%; alcohol use 25.0%.	9
Petry et al. [5]	US NESARC 2001–2002	AUDADIS/DSM-IV	PG (*n* = 195) lifetime prevalence 0.4% (men = 0.6% and women = 0.2%); gamblers associated with higher risk for alcohol use (OR = 6.0), drug use (OR = 4.4), nicotine dependence (OR = 6.7), mood disorders (OR = 4.4), anxiety disorders (OR = 3.9), and personality disorders (OR = 8.3) than the general population.	9
el-Guebaly et al. [19]	Canadian CHS–2.1	PGSI; CIDI/DSM-IV	PG (*n* = 660) annual prevalence 4.1%; moderate/high gambling associated with substance/harmful alcohol use OR = 2.9, mood/anxiety disorders OR = 1.7. Women have a lower risk of gambling.	8
Kessler et al. [6]	US NCS-R 2001–2003	CIDI/DSM-IV	PG lifetime prevalence 0.6%; any DSM-IV comorbid disorder 96.3%; significantly higher ORs for SUD (5.5); mood disorders (3.7; excluding BP-I); anxiety disorders (3.1); impulse control disorders (OR = 2.2).	9
Park et al. [7]	Korea ECA 2006–2007	CIDI/DSM-IV	PG (*n* = 53) lifetime prevalence 0.8%; SUD 69.8% (OR = 6.1); mood disorders 11.6% (OR = 2.9); anxiety disorders 14.0% (OR = 3.9); any DSM-IV mental disorder 79.1% (OR = 7.7).	9
Bischof et al. [8]	Germany PAGE 2009–2011	CIDI/DSM-IV	PG (*n* = 47) lifetime prevalence 0.6%; SUD 87.2% (OR = 4.1); mood disorders 46.8% (OR = 3.1); anxiety disorders 38.3% (OR = 3.8); any DSM-IV mental disorder 93.6% (OR = 5.2).	9
Subramaniam et al. [20]	Singapore MHS 2009–2010	SOGS; CIDI/DSM-IV	PG (*n* = 104) weighted lifetime prevalence 2.7%; any mental disorder associated with PG 38.0%; significantly high ORs for depressive and bipolar disorder, anxiety disorders, alcohol misuse, and nicotine dependence.	9
Assanangkornchai et al. [21]	Thailand NMHS 2013	CIDI/DSM-IV-TR	PG lifetime prevalence 0.9%; alcohol use disorder 57.4% (OR = 5.2); drug use disorder 16.2% (OR = 3.8); nicotine dependence 49.5% (OR = 4.9); mood disorders 10.1% (OR = 9.1); anxiety disorders 5.2% (OR = 2.0); psychotic disorders 14.5% (OR = 2.6); impulse control disorders 14.9% (OR = 6.3).	9
Chen et al. [22]	Telephone interview survey, Macao (CHI)	DSM–5; DASS–21	PD (*n* = 19) prevalence 1.9%; IGD 2.0; comorbid depression 26.3% (OR = 3.3); anxiety 36.8% (OR = 2.0); IGD 21.1% (OR = 5.9).	8
Hakansson et al. [24]	Swedish register survey 2005–2016	ICD–10	PG (*n* = 2,099); anxiety, neurotic, and stress-related disorders (34%), and affective disorders (33%) were the most common comorbid disorders, followed by SUD 25%, personality disorders 10% and psychotic disorders 4%; overall psychiatric comorbidity was 73% (men = 71% and women = 77%).	8
Nicholson et al. [23]	US NESARC 2001–2002	AUDADIS/DSM-IV and DSM–5	DSM–5 GD (*n* = 135) prevalence 1.0%; any alcohol or drug disorders 36.9%, mood disorders 25.6%; anxiety disorders 36.1%; any mental disorder 68.9%. No significant difference in overall and specific Axis I alcohol/drug use, mood, and anxiety disorder rates between DSM-IV and DSM–5 criteria.	9
Gronroos et al. [25]	Finnish register survey 2002–2011	ICD–10	PG (*n* = 3,605); overall psychiatric comorbidity 88.2%; higher for women than for men (92% vs. 87%).	7

Abbreviations: AUDADIS-IV, Alcohol Use Disorder and Associated Disabilities Interview Schedule-DSM-IV Version; BP-I, Bipolar Disorder I; CATI, Computer-Assisted Telephone Interview; CIDI, Composite International Diagnostic Interview; CCHS-2.1, Canadian Community Health Survey-2.1; DASS-21, Depression and Anxiety Stress Scale-21; DIS, Diagnostic Interview Schedule; DSM-IV, Diagnostic and Statistical Manual of Mental Disorders, fourth edition (pathological gambling: 5+ criteria); DSM-5, Diagnostic and Statistical Manual of Mental Disorders, fifth edition (gambling disorder: 4+ criteria); KECA, Korean Epidemiologic Catchment Area study; GAD, Generalized anxiety disorder; GD, DSM-5 gambling disorder; ICD-10, ICD-10 International Classification of Mental and Behavioral disorders; IGD, Internet gaming disorder; OCD, Obsessive compulsive disorder; PAGE, Pathological Gambling and Epidemiology Survey; PG, DSM-IV or ICD-10 pathological gambling; PGSI, Problem Gambling Severity Index (no distinction between problem and pathological gambling); NCS-R, National Comorbidity Survey-Replication; NESARC, National Epidemiologic Survey on Alcohol and Related Conditions; SMHS, Singapore Mental Health Survey; SOGS, South Oaks Gambling Screen; SUD, Substance use disorders; NMHS, National Mental Health Survey.

a JBI checklist for prevalence studies: risk of bias rated as high (0–3) moderate (4–6) or low (7–9).

To assess psychiatric comorbidity of GD, most studies used semi-structured instruments based on DSM-IV [3] and DSM-5 [1] criteria, such as the Alcohol Use Disorder and Associated Disabilities Interview Schedule [28], the Composite International Diagnostic Interview [29], or the Diagnostic Interview Schedule [30]. In addition, two studies used ICD-10 [4] diagnoses collected about in- and outpatients attending mental health services, and another, the Depression and Anxiety Stress Scale. All studies met established methodological standards with scores of 7–9 according to the JBI checklist for prevalence studies [13]. The reported prevalence rates were also weighted or adjusted for sociodemographic factors, and hence JBI score was not tested as a potential moderator in meta-regression.

There were comparable prevalence rates in overall psychiatric morbidity associated with GD between population-based surveys using DSM-IV [5–8, 21] and ICD-10 [24, 25] criteria, with only one outside the range 73–96%, probably because it did not include drug use disorder [20]. Eight studies [5–8, 20–23] also reported that cases with GD had significantly higher ORs for comorbid Axis I disorders (i.e. substance use, mood, anxiety, and impulse control disorders) or personality disorders than the general population.

### Pooled prevalence of mental disorders associated with GD

The weighted average prevalence for any comorbid disorder associated with GD based on a pooled estimate of studies for which information was available was 82.2% (standard error [SE] ± 0.37) (Table 2). Analysis of psychiatric morbidity by the main diagnostic groups revealed similarly high prevalence rates for SUD (34.2%; SE ± 0.29), mood disorders (30.9%; SE ± 0.20), and anxiety disorders (29.9%; SE ± 0.24), followed by personality disorders (14.3%; SE ± 0.30), and psychotic disorders (5.9%; SE ± 0.08).

**Table 2.** Prevalence (%) of ICD/DSM main diagnostic groups of mental disorders associated with gambling disorder

Study	SUD	Psychotic disorders	Mood disorders	Anxiety disorders	Personality disorders	Any disorder
Welte et al. [18]	–	–	–	–	–	–
Petry et al. [5]	–	–	49.6	41.3	60.8	–
el-Guebaly et al. [19]	49.9^a^	–	47.6^c^	–	–	–
Kessler et al. [6]	76.3	–	55.6	60.3	42.3^b^	96.3
Park et al. [7]	69.8	–	11.6	14.0	–	79.1
Bischof et al. [8]	87.2	–	46.8	38.3	–	93.6
Subramaniam et al. [20]	–	–	21.2	1.8	–	38.0
Assanangkornchai et al. [21]	–	14.5	10.1	5.2	14.9^b^	–
Chen et al. [22]	–	–	26.3	36.8	–	–
Hakansson et al. [24]	25.0	4.0	33.0	34.0	10.0	73.0
Nicholson et al. [23]	36.9^a^	–	25.6	36.1	–	68.9
Gronroos et al. [25]	–	–	–	–	–	88.2
**Weighted mean prevalence** (SE)	34.2 (0.29)	5.9 (0.08)	30.9 (0.20)	29.9 (0.24)	14.3 (0.30)	82.2 (0.37)

Abbreviations: SE, standard error; SUD, substance use disorder.

a Substance and alcohol use.

b Impulse control disorders (i.e., attention deficit hyperactivity disorder, oppositional defiant disorder, conduct disorder, and intermittent explosive disorder) excluded from analysis.

c Mood and anxiety disorders.

The weighted mean effect for specific categories was as follows: alcohol use disorder (27.8%; SE ± 0.36), drug use (22.5%; SE ± 0.37), nicotine use (15.8%; SE ± 0.44), major depression (20.3%; SE ± 0.42), bipolar/manic episode (18.6%; SE ± 0.02), GAD (10.5%; SE ± 0.27), and panic disorder (9.8%; SE ± 0.29) (Table 3).

**Table 3.** Prevalence (%) of specific ICD/DSM categories of mental disorders associated with gambling disorder

Study	Alcohol	Drug	Nicotine	Depression	Bipolar/manic	GAD	Panic
Welte et al. [18]	25.0	–	–	–	–	–	
Petry et al. [5]	73.2	38.1	60.4	37.0	27.5	11.5	18.2
el-Guebaly et al. [19]	–	–	–	–	–	–	
Kessler et al. [6]	–	.	63	38.6	17.0^a^	16.6	21.9
Park et al. [7]	–	30.2	34.9	11.6	–	–	–
Bischof et al. [8]	61.7	19.1	68.1	36.2		–	–
Subramaniam et al. [20]	13.1		9.1	13.0	3.5	1.8	
Assanangkornchai et al. [21]	57.4	16.2	49.5	9.1	–	–	3.2
Chen et al. [22]	–	–	–	23.6	–	–	–
Hakansson et al. [24]	17.0	11.8%	0.4	–	–	–	–
Nicholson et al. [23]	35.6	5.2	–	–	–	–	–
Gronroos et al. [25]	–	–	–	–	–	–	–
**Weighted mean prevalence** (SE)	27.8 (0.36)	22.5 (0.37)	15.8 (0.44)	20.3 (0.42)	18.6 (0.02)	10.5 (0.27)	9.8 (0.29)

Abbreviations: GAD, generalized anxiety disorder; SE, standard error.

a Excluded Bipolar disorder I.

### Meta-analysis

**Figure 2.**
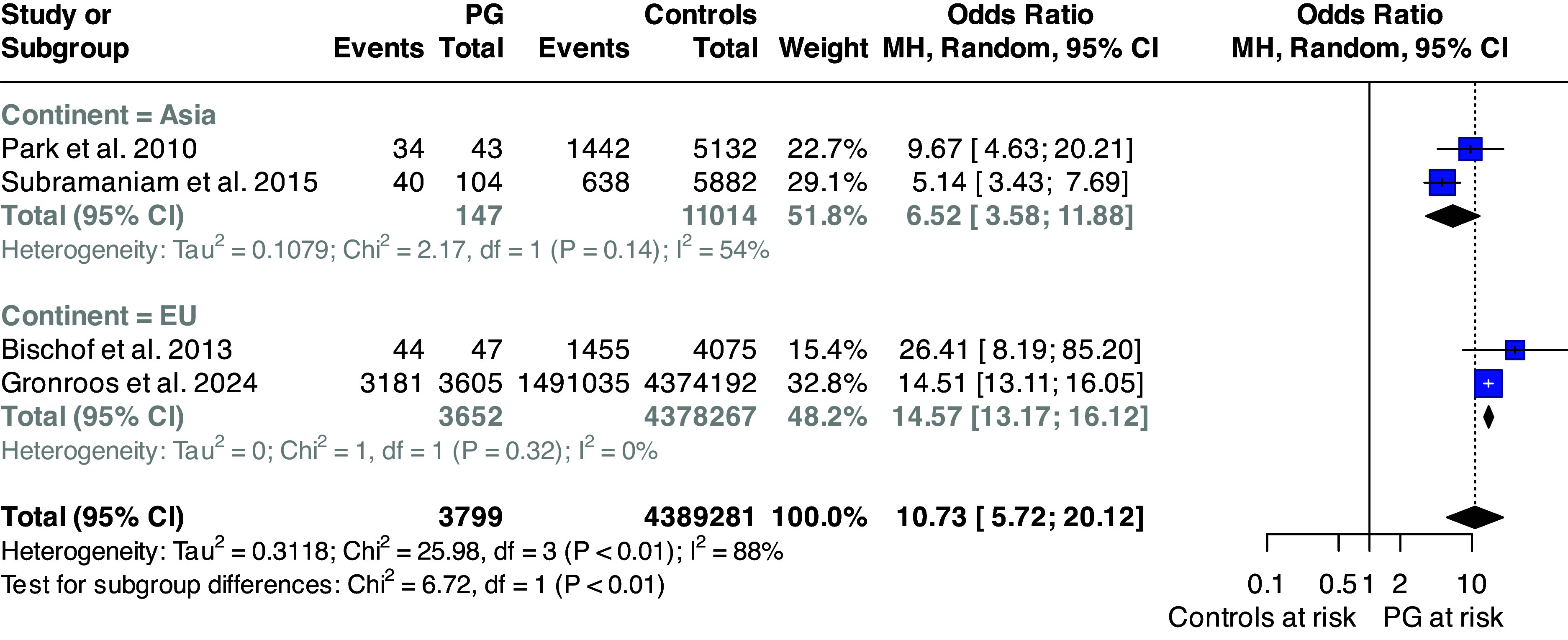
Forest plot of the risk of any mental disorder among individuals with pathological gambling and controls. Abbreviations: PG: pathological gambling; MH: Mantel–Haenszel; df: degree of freedom; 95%CI: 95% confidence interval.

Random effect meta-analysis of pooled studies [7, 8, 20, 25] reporting on overall psychiatric comorbidity suggests that individuals with GD are about 10.73 (95% CI: 5.72; 20.12) times more likely to develop any mental disorder than the general population, with high between-study heterogeneity (*I* ^2^ = 88%; *p* < 0.0001). Subgroup meta-analysis found statistically significant group differences across the continent where the study was performed (*p* = 0.010); the results are displayed in Figure 2.

Although formal testing for publication bias is not recommended when fewer than 10 studies contribute to a pooled estimate, we conducted a qualitative assessment of funnel plot symmetry. This suggested that one study lay outside the funnel area with a smaller effect size (see Supplementary Figure 1). However, the Egger test for funnel plot asymmetry was not statistically significant (*p* = 0.566), supporting a low likelihood of publication bias.

Meta-regression showed that geographical area significantly influenced the pooled estimate, with samples from Europe displaying a higher risk of any mental disorder among individuals with GD compared to Asia (0.91; 95% CI: 0.17; 1.65). Gender also emerged as a significant moderator, as studies with a higher proportion of female participants reported lower ORs (−0.039; 95% CI: −0.066; −0.011).

Subgroup meta-analysis including at least two studies also revealed significantly high ORs for the following conditions associated with GD: SUDs (12.13; 95% CI: 10.36; 14.21), alcohol use disorder (9.89; 95% CI: 6.71; 14.60), drug use disorder (9.73; 95% CI: 2.79; 33.95), nicotine dependence (5.74; 95% CI: 3.31; 9.96), mood disorders (4.01; 95% CI: 3.24; 4.96), and anxiety disorders (3.41; 95% CI: 2.02; 5.78). Additionally, decomposing the mood disorders and anxiety disorders categories into specific diagnoses found similarly high OR among participants with GD: major depressive disorder (3.31; 95% CI: 2.36; 4.65), bipolar disorder (8.24; 95% CI: 6.04; 11.25), and GAD (2.86; 95% CI: 1.89; 4.35). The results are reported in Table 4 and Supplementary Figures 2–10.

**Table 4.** Results of the meta-analysis for each specific mental disorder

Mental disorder	*N* studies	OR (95% CI)	*p*-value	*I* ^2^% (*p*-value)
SUD	3	12.13 (10.36; 14.21)	<0.0001	0% (0.442)
Alcohol use disorder	5	9.89 (6.71; 14.60)	<0.0001	63% (0.027)
Illicit drug use	2	9.73 (2.79; 33.95)	0.0004	89% (0.003)
Nicotine dependence	4	5.74 (3.31; 9.96)	<0.0001	74% (0.010)
Mood disorders^a^	5	4.01 (3.24; 4.96)	<0.0001	22% (0.277)
Anxiety disorders^b^	5	3.41 (2.02; 5.78)	<0.0001	68% (0.014)
MDD	5	3.31 (2.36; 4.65)	<0.0001	35% (0.185)
BD	3	8.24 (6.04; 11.25)	<0.0001	0% (0.537)
GAD	2	2.86 (1.89; 4.35)	<0.0001	0% (0.991)

Abbreviations: BD, bipolar disorder; N, number; NA, not applicable; OR, odds ratio; SUD, substance use disorder; 95%CI, 95% confidence interval.

*Note:* Odds ratios >1 indicate an increased probability of the outcome in the group with pathological gambling.

a Mood disorders include any depressive and bipolar disorder.

b Anxiety disorders include any anxiety and phobic disorder.

Heterogeneity was low for SUD (*I* ^2^ = 0%; *p* = 0.442) and mood disorders (*I* ^2^ = 22%; *p* = 0.277); substantial for alcohol use disorder (*I* ^2^ = 63%; *p* = 0.027), anxiety disorders (*I* ^2^ = 68%; *p* = 0.014), and nicotine dependence (*I* ^2^ = 74%; *p* = 0.010); and high for drug use disorder (*I* ^2^ = 89%; *p* = 0.003). Univariable meta-regression analyses here undertaken found that statistically significant moderators were the year after DSM-5 publication for nicotine use (−1.16; 95% CI: −1.84; −0.482), Europe as the geographical area for mood disorder (0.705; 95% CI: 0.002; 1.41), the percentage of female in the sample (−0.041; 95% CI: −0.065; −0.016), and Europe as the geographical area for anxiety disorders (1.37; 95% CI: 0.601; 2.13).

Full meta-regression results are presented in Supplementary Table 2.

To further explore the potential role of confounding factors in the relationship between GD and mental disorders, we also extracted information on socioeconomic status and physical health conditions when reported in the primary studies. For physical health conditions, only two studies provided comparable data [20, 25], suggesting that individuals with GD had a higher risk of digestive diseases (particularly ulcer and chronic inflammatory bowel disease) and cardiovascular diseases (particularly hypertension) compared to controls. Regarding socioeconomic status, four studies [5, 7, 10, 20] reported relevant measures, three based on income categories [5, 7, 20] and one [22] on the education level. Of these, two studies [7, 22] found a higher proportion of individuals with low socioeconomic status among GD cases than controls, one study reported comparable distributions [5], and one reported a higher prevalence of low socioeconomic status among controls [20]. The meta-analysis of socioeconomic status was, therefore, not statistically significant (OR = 1.52; 95% CI: 0.64; 3.61), as shown in Supplementary Figure 11.

### Sensitivity analyses

Leave-one-out analysis for alcohol use disorder suggested that heterogeneity (*I* ^2^ = 14%) was partly removed, excluding Petry et al. [5], while the effect size slightly increased (OR = 11.3; 95% CI: 8.2; 15.7). For nicotine dependence, excluding Subramaniam et al. [20], which was found to be an outlier, *I* ^2^ dropped to 0% and the effect size increased (OR = 7.4; 95% CI: 5.8; 9.4). For anxiety disorders, both heterogeneity (*I* ^2^ = 10%) and effect size were reduced (OR = 3.0; 95% CI: 2.2; 4.0), excluding Bischop et al. [8]. No outlier was found for the remaining mental disorders associated with GD. The results of leave-one-out analyses are presented in Supplementary Table 3.

## Discussion

This systematic review, complemented by meta-analysis of robust population-based studies, updates and expands the evidence base on the psychiatric morbidity associated with GD in the general population, and marks an advance on earlier reviews in several ways. First, it deals with population studies using ICD-10 and DSM-5 criteria in addition to DSM-IV criteria. Second, it is comprehensive of both large-scale, randomly selected, and treatment-seeking adult representative samples from community mental health services. Since GD is rare [31], and population-based studies are expensive and impractical to undertake on a large scale, it was worth selecting for this review also nationwide register surveys, which usually include a high number of subjects attending secondary care psychiatric services with GD and a broad variety of comorbid mental disorders. Surprisingly, there were only two case-register surveys using ICD-10 criteria [23, 24], and none used ICD-11; therefore, meaningful comparisons with findings of studies based on DSM criteria prove difficult. Third, this review provides pooled prevalence and ORs for diagnostic categories of mental disorders associated with GD, and puts the study of this topic on a new empirical footing.

The resulting picture indicates that: (a) to date, only a few population studies reporting on psychiatric comorbidity of GD have been conducted using ICD-10 [4] or DSM-5 [1] criteria; (b) recently published studies reported prevalence rates and ORs for mental disorders associated with GD similar to those previously reported; (c) the weighted average prevalence of any mental disorder associated with GD based on selected studies was 82.2%, with similarly high rates for SUD (34.2%), mood disorders (30.9%; especially depression and bipolar/manic episode), and anxiety disorders (29.9%; GAD and panic disorder), followed by personality disorders (14.3%) and psychotic disorders (5.9%); (d) meta-analysis of pooled studies using a random effect model reported that people with GD are over 12 times more likely to develop any mental disorder than individuals without either type of disorder; (e) the estimated ORs for psychiatric conditions associated with GD compared with the general population were 5–12 times higher for nicotine dependence, drug use disorder, alcohol use disorder, and SUD, and 3–4 times higher for anxiety and mood disorders, in that order; (f) geographical differences emerged when considering any mental disorder as the outcome, with studies conducted in Europe reporting higher ORs than those from the Asia.

The strong association reported between GD and comorbid mental disorders may, at least partly, be due to changes inherent in modern psychiatric classifications, as they have adopted a multiaxial system listing clinical syndromes and personality separately, and encouraged multiple diagnostics based on operational criteria and temporal cutoffs for each category. The findings of NCS-R [6] revealed that the onset of pathological gambling was preceded in about three in four cases by other DSM-IV mental disorders, especially depression and anxiety disorders, whereas comorbid substance abuse was more likely to occur in the wake of GD, an effect which may result from interaction between common vulnerability and environmental factors [32]. It seems also likely that the diagnostic criteria for DSM-IV pathological gambling tended to overlap with those for substance use, mood, anxiety, and personality disorders [5]. All these issues may cause diagnostic uncertainty and affect estimates of the frequency of comorbid conditions. The only comparative study using DSM-IV and DSM-5 criteria based on NESARC data [23] found no significant difference in prevalence rates of axis I alcohol/drug use, mood, and anxiety disorders associated with GD, while personality disorder was not examined.

Regarding the geographical differences observed in the risk of any mental disorder among individuals with GD, the higher risk found in European compared to Asian samples may reflect the influence of sociocultural factors. Previous research has identified area-level factors, such as economic disadvantage, material and social deprivation, associated with gambling activities [33–35], and these factors have also been associated with poorer mental health outcomes [36, 37]. Considering this background, our findings may appear counterintuitive, given that Europe is generally considered more affluent than Asia. However, the results might have been influenced by the study by Subramaniam et al. [20], conducted in Singapore, which reported a higher prevalence of individuals with low socioeconomic status among controls than GD. Furthermore, area-level factors have been primarily associated with recreational gambling, while their relevance appeared more limited when examined in relation to problematic gambling [38], which is the focus of the current review. In contrast, individual-level factors – particularly male gender – have consistently shown a stronger association with GD than area-level determinants [38]. This is consistent with the results of our meta-regression, in which gender emerged as a significant moderator, with higher proportions of men linked to a greater risk of mental disorders among individuals with GD. Although the small number of studies limited our ability to explore gender-specific rates of psychiatric comorbidity in GD, future research should collect and analyze data stratified by gender and socioeconomic status to generate more robust evidence to inform targeted public health strategies.

It will be rewarding for future research to identify at-risk cases for gambling behavior and undertake prospective studies to investigate clinical and sociodemographic factors involved in the development of comorbid mental disorders. Interestingly, a reanalysis of NESARC data [39] suggested that after 3 years, individuals diagnosed with DSM-5 GD were significantly more likely to develop substance use, mood, and anxiety disorders than nongamblers, an effect correlated to the degree of severity of gambling behavior at intake. On the clinical side, it is desirable that people referred to secondary care psychiatric service for GD are screened not only for addictive behaviors, but also for common mental disorders, such as depression and anxiety, as comorbidity may affect both treatment and outcome.

### Limitations

The findings of the present review need to be viewed cautiously because of several limitations. Although the studies selected achieved established methodological standards according to the JBI checklist [13], population-based register surveys relied on ICD-10 [4] diagnoses collected routinely from patients with GD attending mental health services, rather than using semi-structured interviews and research methods. There is also an incomplete concordance between the ICD-10 [4] and DSM-IV [3] categories of mood, anxiety, habit, and impulse control disorders in terms of clinical syndromes and diagnostic criteria. Additionally, most studies relied on DSM rather than ICD diagnostic criteria, limiting the generalizability of findings across different diagnostic frameworks. Another possible issue hinges on the fact that meta-analysis of psychiatric comorbidity of GD was restricted to main diagnostic groups, such as SUD, mood and anxiety disorders, and no estimate for specific categories – except alcohol, drug, and nicotine use disorder – was provided owing to the paucity of data available. The same consideration applies to potential moderators tested in meta-regression, which varied numerically across comorbid mental disorders. Future studies should focus on collecting data on different diagnoses and stratified by sociodemographic characteristics, such as gender, age, and socioeconomic status, to provide a more granular evidence base on GD and mental disorder comorbidity. Moreover, the risk of publication bias investigation was only exploratory, as it was based on fewer than 10 studies. Lastly, the lack of an adequate number of cases precluded any attempt to determine sex-specific rates of mental disorders associated with GD.

## Supporting information

10.1192/j.eurpsy.2025.10122.sm001 Galeazzi et al. supplementary material Galeazzi et al. supplementary material

## Data Availability

The datasets generated and analyzed during the current study are available from the corresponding author on reasonable request.

## Supplementary material

The supplementary material for this article can be found at http://doi.org/10.1192/j.eurpsy.2025.10122.
