# Supplementary material for: Psychiatric morbidity and gambling disorder: A systematic review and meta-analysis of population-based surveys
Source: Eur Psychiatry. 2025 Oct 23;68(1):e161. doi: 10.1192/j.eurpsy.2025.10122 (PMC12646120; doi:10.1192/j.eurpsy.2025.10122)
Supplement: Galeazzi et al. supplementary material [file S0924933825101223sup001.docx]

**Supplementary Materials**

Supplementary Table 1: search strategy for each database

| Database | Search string | Number of items |
| --- | --- | --- |
| Pubmed | (adult[ti] OR adult[mesh]) AND (gambling[mesh] OR gambling[ti]) AND ("International Classification of Diseases"[Mesh] OR ICD-11 OR ICD-10 OR "Diagnostic and Statistical Manual of Mental Disorders"[Mesh] OR DSM-IV OR DSM-5) AND (epidemiology[ti] OR epidemiology[mesh] OR comorbidity[ti] OR comorbidity[mesh] OR prevalence[ti] OR prevalence[mesh]) Filters: English, from 1993/1/1 - 2025 | 157 |
| Web of Science | gambling (All Fields) and "International Classification of Diseases"OR ICD-11 OR ICD-10 OR "Diagnostic and Statistical Manual of Mental Disorders" OR DSM-IV OR DSM-5 (All Fields) and epidemiology OR comorbidity OR prevalence (All Fields) and adult (All Fields) 1993-2025 | 140 |
| Scopus | (TITLE-ABS-KEY(gambling) AND TITLE-ABS-KEY(adult) AND TITLE-ABS-KEY("International Classification of Diseases" OR icd-11 OR icd-10 OR "Diagnostic and Statistical Manual of Mental Disorders" OR dsm-iv OR dsm-5) AND TITLE-ABS-KEY(epidemiology OR comorbidity OR prevalence)) AND PUBYEAR > 1992 AND ( LIMIT-TO ( LANGUAGE,"English" ) | 390 |

Supplementary Figure 1: Funnel plot of the risk of any mental disorder among individuals with pathological gambling and controls.

Supplementary Figure 2: Forest plot of the risk of substance use disorder among individuals with pathological gambling and controls.

Abbreviations: PG: pathological gambling; MH: Mantel–Haenszel; df: degree of freedom; 95%CI: 95% confidence interval.

Supplementary Figure 3: Forest plot of the risk of alcohol use disorder among individuals with pathological gambling and controls.

Abbreviations: PG: pathological gambling; MH: Mantel–Haenszel; df: degree of freedom; 95%CI: 95% confidence interval.

Supplementary Figure 4: Forest plot of the risk of illicit drug use among individuals with pathological gambling and controls.

Abbreviations: PG: pathological gambling; MH: Mantel–Haenszel; df: degree of freedom; 95%CI: 95% confidence interval.

Supplementary Figure 5: Forest plot of the risk of nicotine use among individuals with pathological gambling and controls.

Abbreviations: PG: pathological gambling; MH: Mantel–Haenszel; df: degree of freedom; 95%CI: 95% confidence interval.

Supplementary Figure 6: Forest plot of the risk of mood disorders among individuals with pathological gambling and controls.

Abbreviations: PG: pathological gambling; MH: Mantel–Haenszel; df: degree of freedom; 95%CI: 95% confidence interval.

Supplementary Figure 7: Forest plot of the risk of anxiety disorders among individuals with pathological gambling and controls.

Abbreviations: PG: pathological gambling; MH: Mantel–Haenszel; df: degree of freedom; 95%CI: 95% confidence interval.

Supplementary Figure 8: Forest plot of the risk of major depressive disorder among individuals with pathological gambling and controls.

Abbreviations: PG: pathological gambling; MH: Mantel–Haenszel; df: degree of freedom; 95%CI: 95% confidence interval.

Supplementary Figure 9: Forest plot of the risk of bipolar disorder among individuals with pathological gambling and controls.

Abbreviations: PG: pathological gambling; MH: Mantel–Haenszel; df: degree of freedom; 95%CI: 95% confidence interval.

Supplementary Figure 10: Forest plot of the risk of generalized anxiety disorder among individuals with pathological gambling and controls.

Abbreviations: PG: pathological gambling; MH: Mantel–Haenszel; df: degree of freedom; 95%CI: 95% confidence interval.

Supplementary Figure 11: Forest plot of the risk of socioeconomic status among individuals with pathological gambling and controls.

Abbreviations: PG: pathological gambling; MH: Mantel–Haenszel; df: degree of freedom; 95%CI: 95% confidence interval.

Supplementary Table 2: meta-regression results

| **Mental disorder** | **Predictor** | **Effect (95%CI)** | **p-value** | **R^2^** |
| --- | --- | --- | --- | --- |
| *SUD* | Age | NA^a^ | NA^a^ | NA^a^ |
|  | % Female^†^ | -0.019 (-0.053; 0.014) | 0.260 | 100% |
|  | Continent Asia/Europe/USA | NA^a^ | NA^a^ | NA^a^ |
|  | Diagnostic manual, DSM/ICD | NA^b^ | NA^b^ | NA^b^ |
|  | Year >2013 | NA^b^ | NA^b^ | NA^b^ |
|  | Sampling, RS/TS | NA^b^ | NA^b^ | NA^b^ |
| *AUD* | Age | NA^a^ | NA^a^ | NA^a^ |
|  | % Female^†^ | -0.011 (-0.051; 0.030) | 0.599 | 0% |
|  | Continent:  Asia  Europe  USA | Ref.  0.330 (-1.18; 1.84)  0.172 (-1.06; 1.40) | Ref.  0.669  0.785 | 0% |
|  | Diagnostic manual, DSM/ICD | NA^b^ | NA^b^ | NA^b^ |
|  | Year:  ≤2013  >2013 | Ref.  -0.126 (-1.25; 1.00) | Ref.  0.826 | 0% |
|  | Sampling, RS/TS | NA^b^ | NA^b^ | NA^b^ |
| *Nicotine dependance* | Age | NA^a^ | NA^a^ | NA^a^ |
|  | % Female^†^ | -0.017 (-0.074; 0.039) | 0.546 | 0% |
|  | Continent:  Asia  Europe  USA | Ref.  0.666 (-1.32; 2.65)  0.554 (-1.36; 2.46) | Ref.  0.511  0.570 | 0% |
|  | Diagnostic manual, DSM/ICD | NA^b^ | NA^b^ | NA^b^ |
|  | Year:  ≤2013  >2013 | Ref.  -1.16 (-1.84; -0.482) | Ref.  0.001 | 100% |
|  | Sampling, RS/TS | NA^b^ | NA^b^ | NA^b^ |
| *Mood disorders* | Age | NA^a^ | NA^a^ | NA^a^ |
|  | % Female^†^ | -0.020 (-0.043; 0.003) | 0.095 | 100% |
|  | Continent:  Asia  Europe  USA | Ref.  0.705 (0.002; 1.41)  0.278 (-0.208; 0.764) | Ref.  0.050  0.262 | 100% |
|  | Diagnostic manual, DSM/ICD | NA^b^ | NA^b^ | NA^b^ |
|  | Year:  ≤2013  >2013 | Ref.  -0.223 (-0.788; 0.342) | Ref.  0.439 | 0% |
|  | Sampling, RS/TS | NA^b^ | NA^b^ | NA^b^ |
| *Anxiety disorders* | Age | NA^a^ | NA^a^ | NA^a^ |
|  | % Female^†^ | -0.041 (-0.065; -0.016) | 0.001 | 98.8% |
|  | Continent:  Asia  Europe  USA | Ref.  1.37 (0.601; 2.13)  0.401 (-0.150; 0.952) | Ref.  0.001  0.154 | 100% |
|  | Diagnostic manual, DSM/ICD | NA^b^ | NA^b^ | NA^b^ |
|  | Year:  ≤2013  >2013 | Ref.  -0.656 (-1.70; 0.385) | Ref.  0.217 | 17.32% |
|  | Sampling, RS/TS | NA^b^ | NA^b^ | NA^b^ |
| *Any mental disorder* | Age | NA^a^ | NA^a^ | NA^a^ |
|  | % Female^†^ | -0.039 (-0.066; -0.011) | 0.006 | 83.54% |
|  | Continent:  Asia  Europe | Ref.  0.907 (0.168; 1.65) | Ref.  0.016 | 75.83% |
|  | Diagnostic manual:  DSM  ICD | Ref.  0.225 (-1.21; 1.66) | Ref.  0.758 | 0% |
|  | Year:  ≤2013  >2013 | Ref.  -0.584 (-1.64; 0.470) | Ref.  0.277 | 0% |
|  | Sampling:  RS  TS | Ref.  0.225 (-1.21; 1.66) | Ref.  0.758 | 0% |
| *Illicit drug use* | Age | NA^a^ | NA^a^ | NA^a^ |
|  | % Female^†^ | NA^a^ | NA^a^ | NA^a^ |
|  | Continent: Asia/Europe/USA | NA^a^ | NA^a^ | NA^a^ |
|  | Diagnostic manual, DSM/ICD | NA^b^ | NA^b^ | NA^b^ |
|  | Year >2013 | NA^b^ | NA^b^ | NA^b^ |
|  | Sampling, RS/TS | NA^b^ | NA^b^ | NA^b^ |

Abbreviations: AUD: alcohol use disorder; DSM: diagnostic and statistical manual of mental disorders; ICD: International Classification of Disease; SUD: substance use disorder; NA: not applicable due to insufficient number of information available from the included studies or due to same characteristics of all the studies contributing to the pooled estimate; USA: United States of America; Ref.: reference group; RS: random sampling; TS: treatment seeking sampling; 95%CI: 95% confidence interval.

Legend: the effects are presented as log-odds. Positive values indicate an increased probability of the outcome in the group with pathological gambling.

^†^ Sex assigned at birth

^a^ Not applicable due to insufficient number of information available from the included studies.

^b^ Not applicable due to same characteristics of all the studies contributing to the pooled estimate.

Supplementary Table 3: leave-one-out analysis

| **Mental disorder** | **Author, year** | **OR (95%CI)** | **p-value** | **I^2^%** |
| --- | --- | --- | --- | --- |
| *SUD* | Bischof et al., 2013 | 11.93 (10.16; 14.01) | <0.001 | 0% |
|  | el-Guebaly et al., 2006 | 13.09 (6.66; 25.7) | <0.001 | 38% |
|  | Park et al., 2010 | 12.74 (9.47; 17.16) | <0.001 | 16% |
| *AUD* | Bischof et al., 2013 | 9.49 (5.92; 15.21) | <0.001 | 69% |
|  | Park et al., 2010 | 10.42 (6.30; 17.25) | <0.001 | 73% |
|  | Petry et al., 2005 | 11.31 (8.17; 15.67) | <0.001 | 14% |
|  | Subramaniam et al., 2015 | 10.42 (6.18; 17.55) | <0.001 | 72% |
|  | Welte et al., 2001 | 8.42 (6.15; 11.54) | <0.001 | 41% |
| *Nicotine dependance* | Bischof et al., 2013 | 5.12 (2.47; 10.60) | <0.001 | 83% |
|  | Park et al., 2010 | 5.25 (2.48; 11.12) | <0.001 | 85% |
|  | Petry et al., 2005 | 5.19 (2.35; 11.46) | <0.001 | 79% |
|  | Subramaniam et al., 2015 | 7.37 (5.79; 9.39) | <0.001 | 0% |
| *Mood disorders* | Bischof et al., 2013 | 3.73 (2.97; 4.70) | <0.001 | 0% |
|  | Chen et al., 2018 | 4.07 (3.28; 5.07) | <0.001 | 0% |
|  | Park et al., 2010 | 4.16 (3.34; 5.18) | <0.001 | 0% |
|  | Petry et al., 2005 | 3.70 (2.33; 5.89) | <0.001 | 42% |
|  | Subramaniam et al., 2015 | 4.01 (2.78; 5.79) | <0.001 | 33% |
| *Anxiety disorders* | Bischof et al., 2013 | 2.96 (2.23; 3.95) | <0.001 | 10% |
|  | Chen et al., 2018 | 3.67 (1.99; 6.76) | <0.001 | 78% |
|  | Park et al., 2010 | 3.63 (1.94; 6.81) | <0.001 | 78% |
|  | Petry et al., 2005 | 3.36 (1.63; 6.93) | 0.001 | 72% |
|  | Subramaniam et al., 2015 | 3.78 (2.04; 6.98) | <0.001 | 74% |
| *Any mental disorder* | Bischof et al., 2013 | 9.12 (4.76; 17.48) | <0.001 | 89% |
|  | Gronroos et al., 2024 | 9.57 (4.01; 22.85) | <0.001 | 77% |
|  | Park et al., 2010 | 11.40 (4.68; 27.76) | <0.001 | 93% |
|  | Subramaniam et al., 2015 | 14.46 (13.08; 15.98) | <0.001 | 0% |
| *Illicit drug use* | Bischof et al., 2013 | 5.41 (4.05; 7.24) | <0.001 | 0% |
|  | Petry et al., 2005 | 19.46 (8.93; 42.4) | <0.001 | 0% |

Abbreviations: AUD: alcohol use disorder; SUD: substance use disorder; OR: odds ratio; 95%CI: 95% confidence interval.

Legend: Odds ratios >1 indicate an increased probability of the outcome in the group with pathological gambling.
